# Chondroblastoma of the patella with pathological fracture in an adolescent: a case report

**DOI:** 10.1186/s12957-019-1760-z

**Published:** 2019-12-12

**Authors:** Yun Lang, Qing Yu, Yuehong Liu, Lejin Yang

**Affiliations:** 1 Department of Orthopedics, People’s Hospital of Deyang City, Taishan North Road 173#, Deyang, 618000 Sichuan Province People’s Republic of China; 2 Department of Pathology, People’s Hospital of Deyang City, Taishan North Road 173#, Deyang, 618000 Sichuan Province People’s Republic of China

**Keywords:** Chondroblastoma, Patella, Pathological fracture

## Abstract

**Background:**

Chondroblastoma is a rare primary bone tumor of young people that generally occurs in the epiphyseal plate of long bones. To date, only 13 cases of patella with pathological fracture in chondroblastoma have been previously published.

**Case presentation:**

A 15-year-old male patient presented with acute pain in the left knee after an injury occurred while playing basketball. Plain radiographs and computed tomography showed a pathological fracture of the left patella with an osteolytic lesion (1.5 × 2 × 3 cm). Magnetic resonance imaging revealed an expansile lesion within the patella with a slightly high signal on the T1-weighted image, a high signal on the T2-weighted image and soft tissue swelling in front of the patella. A ^m99^Tc bone scintigraphy revealed moderate uptake. The preoperative diagnosis was chondroblastoma. This patient underwent intralesional meticulous extended curettage, adjuvant high-speed burr, 95% alcohol and electrotome treatment, autogenous iliac crest bone grafting, and internal fixation. A postoperative pathological diagnosis was chondroblastoma. The patient’s function was satisfactory, and there was no sign of tumor recurrence. The internal fixator was good, with no loosening or migration observed at the last follow-up at 20 months after surgery.

**Conclusions:**

Rarely, chondroblastoma of the patella can present with acute pain due to pathological fracture. We present the 14th such case in the literature to associate patellar chondroblastoma with pathological fracture. The patient was treated with curettage, inactivation, autogenous bone grafting, and internal fixation. A satisfactory therapeutic effect was obtained. This case may be beneficial to the diagnosis and treatment of chondroblastoma patella.

## Background

Chondroblastomas (CBs) are benign cartilaginous lesions that account for less than 1% of all bone tumors and less than 3% of benign bone tumors [1, 2]. CBs usually developed in adolescents and young adults, and men are more commonly affected than women. CB arises from a secondary ossification center in epiphyseal plates and apophyses of a long bone, such as the proximal tibia or femur, distal femur, and proximal humerus [3–5]. The patella is an infrequent site [6].

Cohen described the first case of patellar CB in 1963 [7]. To the best of our knowledge, CB in the patella with pathological fracture is rare, with only case reports being reported in the English language literature [2, 8–13]. However, 13 cases of patella CB with pathological fracture were reported in the previous literature. This report is the 14th such case.

## Case presentation

A 15-year-old male patient presented to the emergency department for acute pain of the left knee after trauma resulting from a direct hit while playing basketball. The patient denied having any history of chills, fever, or weight changes. His family history was insignificant for unspecified malignancy in both parents.

In the physical examination, there was direct tenderness on the left patella. The left anterior patella showed edema. However, there were no palpable masses or local heat. The range of motion (ROM) of the left knee was restricted, and the left lower limb could not bear weight. There were no abnormal neurological findings. There was no external shortening or rotation of the leg. The lateral stress test of the knee joint, McMurray’s test and posterior-anterior drawer test were negative.

**Fig. 1:**
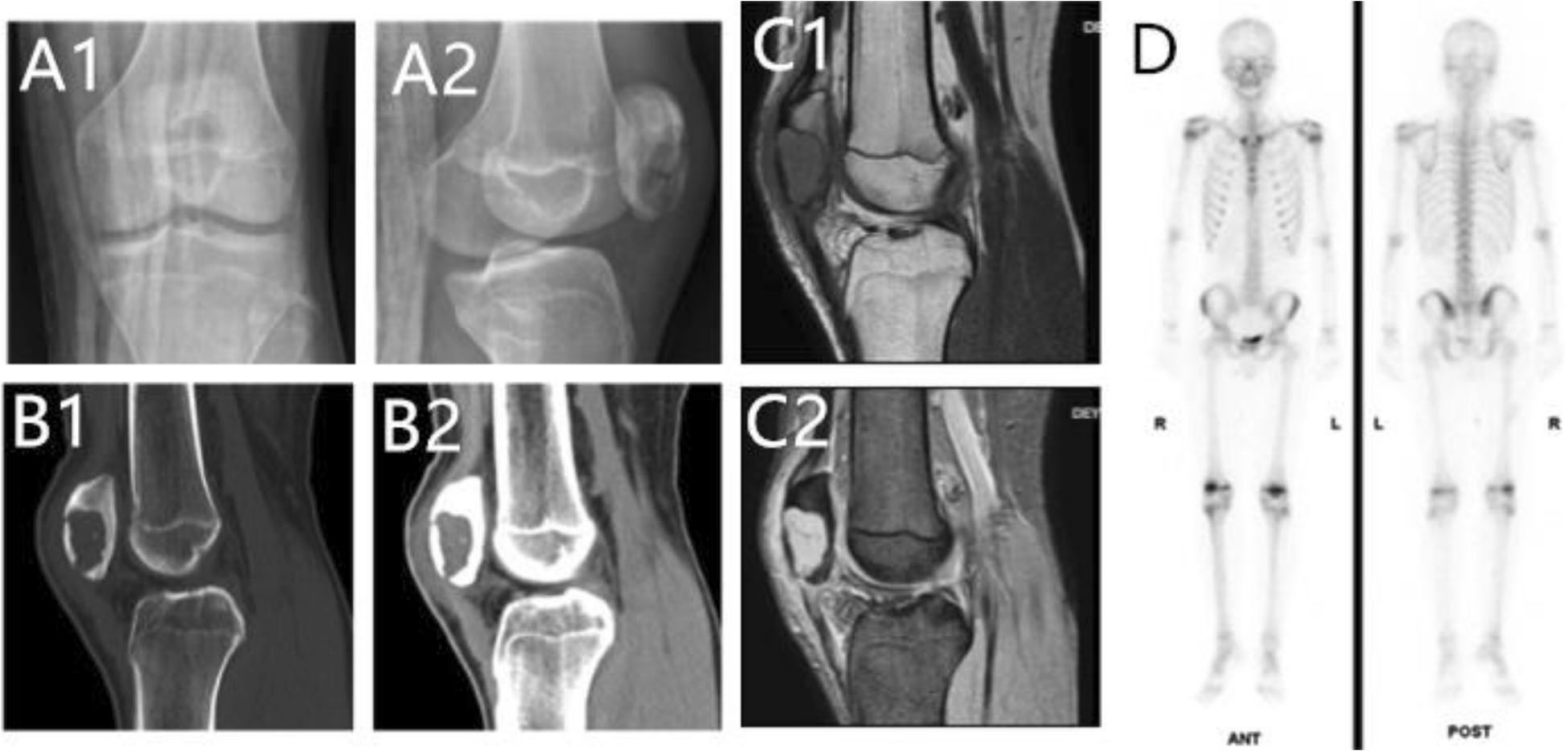
Images of the patient who had pathological fracture of his left patella. **A1**, **A2** Anteroposterior and lateral views showed a well-demarcated, eccentric and osteolytic lesion with pathological fracture of the left patella. **B1**, **B2** CT scan revealed a stage 2 patella lesion (1.5 × 2 × 3 cm) with a thinned cortex, a clear boundary and pathological fracture. Calcifications inside the lesion are shown very well in **B2**. The lesion did not break through the articular cartilage and did not invade the adjacent femur or tibia. **C1**, **C2** MRI showed that the lesion appeared as slightly intense on T1-weighted images (**C1**, through the knee in sagittal planes) and intense on T2-weighted images (**C2**, through the knee in sagittal planes). The soft tissue showed edema at the anterior of the patella. **D** Whole-body bone scan of the patient. A bone scan showed moderate accumulation of the radiopharmaceutical agent in the left and right patella without strong accumulation in other parts of the body

Laboratory testing included a comprehensive metabolic panel, tumor markers and complete blood cell count results within the normal range, except for an alkaline phosphatase level of 153 U/L (reference range, 40–150). Radiography of the knee (Fig. 1A) revealed a well-demarcated, eccentric, and osteolytic lesion with pathological fracture of the left patella by a uniformly radiolucent and expansile process that did not involve any adjacent osseous structures. A computed tomography (CT) scan showed a thin sclerotic rim with fine matrix calcification within the lesion (1.5 × 2 × 3 cm) (Fig. 1B). MRI (Fig. 1C) demonstrated pathological fracture and a solitary solid enhancing tumor that caused more than half of the destruction of the left patella, with expansion of the osseous margin. In addition, the case showed intensity on T2-weighted images and slight intensity on T1-weighted images. The soft tissue showed edema in the anterior of the patella. A whole-body bone scan with technetium-99 m-methylene-diphosphonate (^99m^Tc-MDP) revealed moderate uptake in the left and right patella (Fig. 1D) without strong accumulation in other parts of the body. However, the right knee was painless, its ROM was not restricted, and its patella was normal on X-ray. Chest radiography showed no abnormal changes. The lesion was stage 2 according to the Enneking system for staging benign and malignant musculoskeletal tumors of Patrick et al. [14].

Considering the appearance and location of this tumor, a primary diagnosis of benign tumor was made. Among CB, giant cell tumor, aneurysmal bone cyst, giant cell reparative granuloma, chondromyxoid fibroma, and brown tumor of hyperparathyroidism, the most likely diagnosis was CB.

**Fig. 2:**
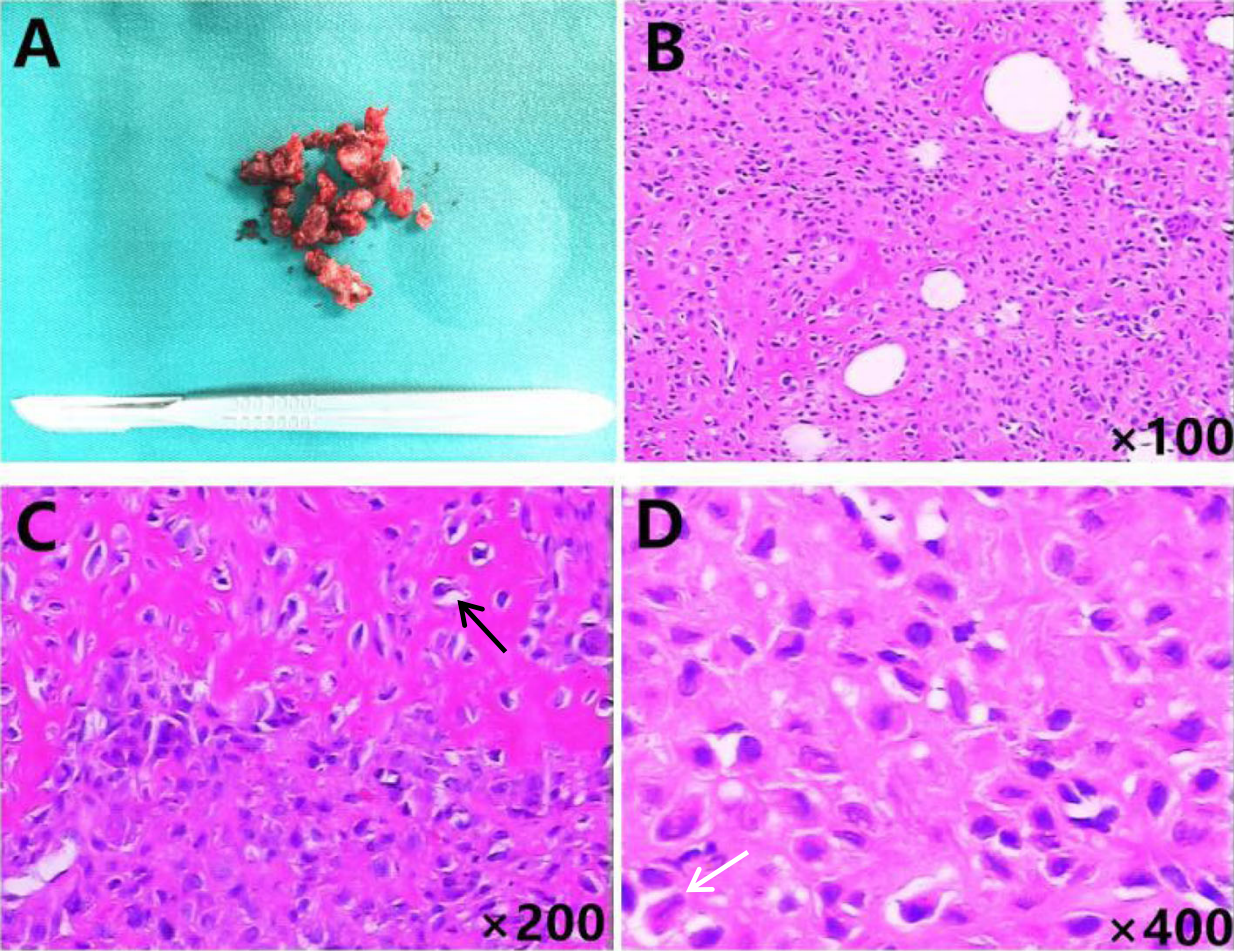
Histological appearance of the tumor (**b**, **c**, **d** H&E). **a** The gross appearance of the tissue specimen showed it contained a mixture of soft, friable, yellow-white material and hemorrhage. **b** High-power photomicrograph showing round or polyhedral cells with oval nuclei in the areas of proliferation embedded in a chondroid matrix (× 100). **c** Focally, a clear cell change (black arrow) could be seen (× 200). **d** The nucleus was centrally placed, and a central, longitudinal nuclear groove (“coffee bean” nucleus) (white arrow) can be seen (× 400)

Preoperative contraindications were excluded. The operation was performed in the People’s Hospital of Deyang City, Sichuan University, by bone tumor surgeons (Drs. Yuehong Liu and Lejin Yang). The patient was placed under intravenous general anesthesia with the use of a tourniquet ischemia. The left knee was covered with disinfecting solution and paving sterile drapes and positioned to expose the surgical field. A longitudinal midline 6-cm incision was formed overlying the left patella. The tissue layers including the skin, subcutaneous tissue, and superficial and deep fascia, was separated. A thin and broken periosteal shell was exposed, and the surrounding soft tissue was not invaded by the tumor. The broken periosteal shell was removed with the attached tumor tissue. The bone window (2.5 × 3 cm) was located the anterior and inferior of the patella. Thorough curettage of the visualized tumor tissues was performed. A high-speed burr was used to grind the tumor border and cancellous bone for 5 mm around the lesion edge. The tumor cavity was inactivated by 95% alcohol for 20 min. Next, we used an electrotome to burn the cavity and washed the cavity deliberately with sterilizing water. The patella was subsequently filled with autogenous iliac cancellous bone and structural cortical bone block to reconstruct cortical defects. To prevent further fracture, tension band wiring fixation of the patella was implemented and carefully closed and repaired with interrupted sutures of each layer of soft tissue. The total loss of blood was ~ 100 ml, and no blood transfusion was performed during the hospitalization. The specimen contained a mixture of soft, friable, yellow-white material, and hemorrhage (Fig. 2A). Intraoperative tissues were sent for pathological examination.

Histological examination showed round or polyhedral cells with oval nuclei in the areas of proliferation embedded in a chondroid matrix (Fig. 2B). However, focally, a clear cell change was observed (Fig. 2C). The nucleus was centrally placed, and a central, longitudinal nuclear groove (“coffee bean” nucleus) was observed (Fig. 2D). Some nucleoli were small. Histologically, CB was confirmed.

**Fig. 3:**
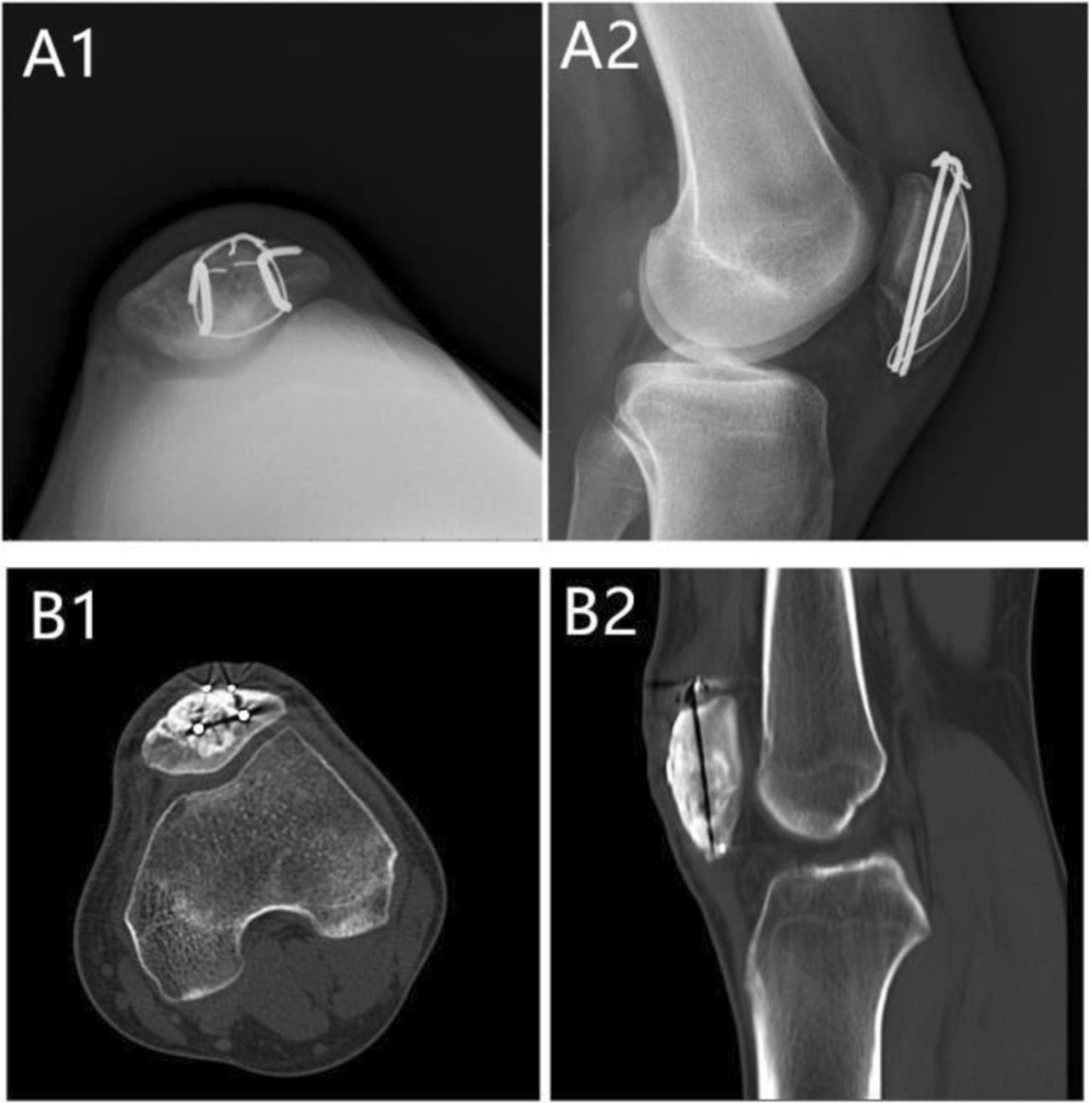
X-ray and CT scan obtained at 21 months after the operation. **A**, **B A1** and **A2** Axial images of the patella and lateral X-ray films of the knee joint showed that the grafting bone had been completely incorporated with the host bone, and the fixation showed no looseness or rupture. **B1** and **B2** Cross and sagittal CT scans revealed bony union

Antibiotics were administered for 24 h (cefazolin 1 g iv), and the rehabilitation program that was started the second day following surgery consisted of muscle strength, joint mobilization training, and partial weight-bearing, as tolerated. The wound healed to grade A. Follow-up occurred at 1, 2, 3, 6, 9, and 12 months and then every 6 months after surgery. At the same time, the patient was followed up with plain tomography, and a CT scan was performed to observe the healing of the grafted bone and tumor recurrence. At 2 months after the operation, the patient could go to school and participate in some simple sports activities. The functional outcome was evaluated with the Pediatric Outcomes Data Collection Instrument (PODCI) questionnaire developed by the Pediatric Orthopaedic Society of North America (POSNA) [15]. At the last follow-up at 21 months after surgery, there was no evidence of local recurrence or distance metastasis (Fig. 3). The internal fixation showed no loosening or migration. The global PODCI score was 100 points. The patient’s sports and physical functioning domain score was 100 points.

## Discussion

CB is a rare primary benign cartilaginous neoplasm of bone that accounts for less than 1% of all bone tumors and less than 3% of benign bone tumors [1, 2, 4]. CB arises from a secondary ossification center in epiphyseal plates and apophyses of a long bone in patients between 10 and 30 years old ( 70% occur during active epiphyseal plate growth) and is more prevalent in males (2:1) [2–4]. This tumor was described as a “calcifying giant cell tumor” by Ewing [16] until Jaffe and Lichtenstein described the entity CB in 1943 [17].

The patella is an uncommon location for bone tumor occurrence and development. Several studies have reported patella tumors [2, 11–13, 18]. Most tumors of the patella are benign. The most common benign lesions are CB, giant cell tumor, and aneurysmal bone cysts [2, 18]. Malignant lesions include metastasis, osteosarcoma, lymphoma, and hemangioendothelioma. The most common tumor-like lesion is a brown tumor in hyperparathyroidism.

The patella is the biggest sesamoid bone inside the human body and is located at the inside of the quadriceps tendon. It appears in the third month of gestation from a cartilaginous precursor that ossifies at approximately 3 years of age [19]. It is an important module in the knee extension system. Most CBs occur in the epiphysis of a skeletally immature long bone, such as the proximal tibia or femur, distal femur, and proximal humerus, and this explains their low incidence in the patella [3–5]. Kurt et al. found that CBs might involve nontubular bones such as the craniofacial skeleton or the hands and feet or flat bone in older individuals. They suggested that CBs can arise in the pelvis, ribs, sternum, clavicle, vertebrae, and patella [20]. CBs arising from the flat bones may extend to an apophysis or an articular surface.

Cohen and Cahen reported the first case of CB in the patella [12]. To our knowledge, cases of patella CBs with pathological fracture are rare. Only three such cases were described thoroughly in case reports including diagnosis, radiology, histology, treatment, and follow-up [8–10]. The other cases were only mentioned and not described in detail [2, 11–13]. We summarize the relevant information, including imaging, diagnosis, and treatments, in Table 1 of this article. Moser et al. [12] described 2 cases, and Casadei et al. [13] described 6 cases with radiologic characteristics of patella CBs with pathological fracture but did not provide their treatment information. In our case presentation, we describe this patient’s images, histology, treatment, and follow-up information. And this case is the 14th such case.

**Table 1:** Review of the current literature of patella CB with pathological fracture

Author	Patients number	Pathological fracture	Imaging	Diagnosis	Treatment	Outcome	PDF available
James et al. [9]	1	1	-	CB	-	-	-
Moser et al. [12]	16	2	-	CB	Not mentioned	Not mentioned	Yes
Wolfe et al. [8]	1	1	-	CB + ABC	Not mentioned	Not mentioned	Only abstract available
Gudi et al. [10]	1	1	RP	CB	Patellectomy	Satisfied	Yes
Bhagat et al. [11]	2	1	-	CB	Curettage	Satisfied	Only abstract available
Singh et al. [2]	9	1	R	CB	Not mentioned	Not mentioned	Yes
Casadei et al. [13]	8	6	RC	CB	Not mentioned	Not mentioned	Yes
This case	1	1	RMCSP	CB	Curettage + autogenous bone grafting + internal fixation	Satisfied	Yes

*CB*, chondroblastoma; *R*, radiograph; *M*, magnetic resonance imaging; *C*, computed tomography; *S*, skeletal scintigram; *P*, pathological section of tumor

The symptoms in many reports include local pain, swelling, joint stiffness and/or effusion, and the development of a limp [3–6, 21–23]. Physical examination may reveal swelling, local tenderness, joint effusion, or local mass. However, rare cases had a history of injury. On X-ray, CB presents as well-defined and eccentric with a thin sclerotic rim of a long bone. CT demonstrates similar findings, although cortical destruction is more clear. Pathologic fracture occurs in a small number of cases. MRI may show that extensive edema and/or periosteal reaction may be present surrounding the lesion. MRI demonstrates variable or heterogeneously high signal intensities on T2-weighted sequences. A ^99m^Tc-MDP bone scan will reveal moderate to intense uptake around the periphery of the lesion [3, 8, 17, 19, 21, 22] and revealed moderate uptake in the left and right patella in our patient. However, there were no signs or symptoms of discomfort in the right knee, and the right knee was normal on an X-ray. These features help distinguish CB from other lesions that may be found in the patella, such as enchondroma, giant cell tumor, aneurysmal bone cyst, chondromyxoid fibroma, nonossifying fibroma, solitary bone cyst, and osteoid osteoma [12, 18, 19].

Histologically, CB is characterized by round or polyhedral chondroblasts with a central, longitudinal nuclear groove (“coffee bean” nucleus). In the majority of lesions, islands of cartilage or chondroid matrix can be found [6, 21–23]. Edel et al. considered that the microscopic appearance of CB can be distinguished from that of other cartillage-containing tumors, such as giant cell tumors of aneurysmal bone cysts [24]. In rare cases, tumor necrosis, cortical breakthrough, vascular invasion, and soft issue invasion can be present [21]. Cozzolino et al. described the cytopathologic features of CB based on fine-needle aspiration smears [25]. Smears showed a variable cellularity represented by a double cell population of mononucleated and multinucleated cells in a noninflammatory background. Nuclei typically showed deep incisures, resulting in a “coffee-bean” appearance. Grooves were always present in their series. In the case of Gudi et al. [10], histopathology revealed trabecular bone admixed with proliferating chondroid tissue at places admixed with myxoid and fibrous tissue with focal areas of calcification. Our case presented the abovementioned typical pathological features of round or polyhedral tumor cells with oval nuclei in a chondroid matrix and a “coffee-bean” appearance.

The primary treatment for stage 1–2 lesions is surgery, including the following steps. First, thorough intralesional curettage was performed to remove all the visualized tumor tissues, and a high-speed burr was used to grind and resect the tumor border and the bone around the lesion. Second, 95% alcohol, phenol, liquid nitrogen, or electrotome were used to eliminate the remaining tumor cells in the cavity. Furthermore, autologous or homologous bone was harvested to reconstruct the resultant bone defects. Finally, for some cases, internal fixation was used to prevent future pathological fractures [2, 4, 6, 18, 19, 23]. This patient underwent treatment via the above steps and obtained a satisfactory tumor prognosis and functional effect. Erickson et al. found that percutaneous radio-frequency heat ablation is a safe and effective treatment for chondroblastoma [3, 10]. Patellectomy has been described in the early literature [10, 19], and Gudi et al. [10] reported that the patient had a full range of movements at the end of 2 years; however, it was not proposed as the first-line treatment for CB. Radiation therapy is rarely used in conjunction with surgery either preoperatively or postoperatively. Chemotherapy has not been indicated in the treatment of patients with CB [8, 21]. Postoperatively, the patient was encouraged to perform rehabilitation exercises, such as joint mobilization and static quadricep exercise, under the guidance of a doctor as this was necessary for the patient to recover normal joint activities [18].

Based on the previous experience mentioned above, we selected the appropriate treatment plan suitable for the patient. The patient underwent curettage and inactivation of the lesion. Reconstruction involved augmentation by autogeneous cancellous bone, a structural cortical bone block, and tension band wiring fixation. At 21 months after the surgery, the global PODCI score sufficiently showed satisfactory functional outcomes. After the lesion had healed well, the internal fixation was not considered for removal. Longer follow-up is needed to monitor other complications and tumor outcomes in this case.

## Conclusions

CB of the patella rarely presents with acute pain due to pathological fracture. We present the 14th case reported in the English literature that associates patellar chondroblastoma with pathological fracture; the patient was treated with curettage, inactivation, autogenous bone graft, and internal fixation. A satisfactory therapeutic effect was obtained. This case may be beneficial to the diagnosis and treatment of CB in patella.

## Data Availability

The authors declare that all data supporting the findings of this study are available within the article.

## Abbreviations

^99m^Tc-MDP: Technetium-99 m-methyle-nediphosphonate
CB: Chondroblastoma
CT: Computed tomography
H&E: Hematoxylin eosin
MRI: Magnetic resonance imaging
PODCI: Pediatric Outcomes Data Collection Instrument
POSNA: Pediatric Orthopaedic Society of North America
ROM: Range of motion
